# Application of Multimodal and Molecular Imaging Techniques in the Detection of Choroidal Melanomas

**DOI:** 10.3389/fonc.2020.617868

**Published:** 2021-02-01

**Authors:** Xuying Li, Lixiang Wang, Li Zhang, Fei Tang, Xin Wei

**Affiliations:** ^1^ Department of Ophthalmology, West China Hospital, Sichuan University, Chengdu, China; ^2^ Department of Ophthalmology, ShangjinNanfu Hospital, Chengdu, China

**Keywords:** choroidal melanoma, multimodal imaging, diagnosis, staging, positron-emission tomography/computed tomography scan

## Abstract

Choroidal melanomas are the most common ocular malignant tumors worldwide. The onset of such tumors is insidious, such that affected patients often have no pain or obvious discomfort during early stages. Notably, enucleation is required for patients with a severe choroidal melanoma, which can seriously impact their quality of life. Moreover, choroidal melanomas metastasize early, often to the liver; this eventually causes affected patients to die of liver failure. Therefore, early diagnosis of choroidal melanomas is extremely important. Unfortunately, an early choroidal melanoma is easily confused with a choroidal nevus, which is the most common benign tumor of the eye and does not often require surgical treatment. This review discusses recent advances in the use of multimodal and molecular imaging to identify choroidal melanomas and choroidal nevi, detect early metastasis, and diagnose patients with choroidal melanomas.

## Introduction

Choroidal melanomas are the most common intraocular malignant tumors worldwide, as well as the second most common type of malignant melanoma. However, the current consensus is that choroidal melanomas and cutaneous melanomas are different types of tumors (1). Thus, their causes, pathogenesis, diagnosis, treatment, and prognosis are quite different (2). Choroidal melanomas can originate from choroidal nevi, which are the most common benign ocular tumors and typically do not require surgical treatment. Notably, 6–10% of patients with a choroidal melanoma have a second primary tumor (3). Furthermore, patients with an advanced unilateral retinoblastoma may have isolated choroidal melanocytosis in the contralateral eye, which can progress to a choroidal melanoma (4, 5).

Choroidal melanomas have an insidious onset and often do not result in pain or obvious discomfort, until they cause inflammation, neovascular glaucoma, or ocular extension (3). Nevertheless, patients with a severe choroidal melanoma require enucleation, which can seriously impact their quality of life. In the past decade, radiotherapy has gradually become the first-line treatment for such patients (6); this treatment modality includes proton beam and plaque brachytherapy (7). However, compared with enucleation, the 5-year survival rate of radiotherapy has not significantly improved (8).

The prognosis of a choroidal melanoma is closely linked with its cytogenetic type and histological grade, which is a distinguishing feature from other cancers (9). The haplotype of chromosome 3 and amplification of chromosome 8 are significantly associated with tumor metastasis, consistent with the American Joint Committee on Cancer (AJCC) staging of the tumor. In addition, a combination of AJCC staging and cytogenetic status can provide greater accuracy than separate assessment methods in predicting patient prognosis (10). At the genetic level, mutations in *BAP1*, *EIF1AX*, *SF3B1* and other genes have been shown to affect patient prognosis; specifically, patients with *EIF1AX* mutations have relatively more positive prognoses, while patients with *SF3B1* mutations are more prone to have advanced metastasis. Furthermore, absence of the tumor suppressor gene *BAP1* will lead to tumor metastasis, thus significantly reducing the survival rate among affected patients (11–13). Choroidal melanomas have been classified into two types on the basis of the gene expression profile: I (low-risk) and II (high-risk) (14). For patients with type II uveal melanoma, the prognosis is not influenced by interventions, but has a strong relationship with the largest basal diameter (LBD); LBD < 12 mm is associated with better prognosis (14, 15). Many prognostic prediction models have been developed, including the Liverpool Uveal Melanoma Prognosticator Online; this model combines tumor histology (e.g., LBD), genetic status (e.g., chromosome 3 monosomy), and other factors to predict all-cause mortality and support personalized treatment (16–18).

Metastasis reportedly affects 50% of patients with uveal melanomas (1). The most common route of choroidal melanoma metastasis is through blood to the liver (19); this event occurs early in the progression of disease. Some results have implied that early metastasis occurs 5 years before the diagnosis and treatment of a choroidal melanoma (3). Other studies have suggested that metastasis is inevitable for patients with choroidal melanomas (20); there remains no optimal scheme or evidence for the treatment of metastatic uveal melanomas (1).

Because of the insidious onset and poor prognosis of choroidal melanomas, early diagnosis of affected patients is extremely important (14). The gold standard for tumor diagnosis is a biopsy and subsequent pathological examination. However, fine-needle aspiration biopsy of the vitreous body in an eye with a choroidal melanoma can lead to seeding of the ciliary body and sclera (21). Therefore, fine needle aspiration biopsy is not an ideal diagnostic method for patients with a suspected choroidal melanoma. Furthermore, early non-invasive diagnosis is important for timely detection of choroidal melanomas and prediction of patient prognosis.

This review focuses on the important roles of multimodal and molecular imaging (e.g., positron emission tomography/computed tomography [PET/CT]) in the identification and monitoring of choroidal nevi, early detection of choroidal melanomas, and recognition of metastasis.

## Identification and Monitoring of Choroid Nevus

According to the Collaborative Ocular Melanoma Study classification, a choroidal nevus constitutes a choroidal melanocytic lesion with LBD ≤ 5 mm and thickness ≤ 1 mm (22). Most instances of choroidal nevus (91%) occur in the posterior portion of the eye (22). Histologically, a nevus is composed of benign cells (i.e., atypical melanocytes). A choroidal nevus is not a congenital condition and most often is acquired (23). Some studies indicate that a high estrogen level and high body mass index are important risk factors for choroidal nevi (24). Furthermore, Singh et al. suggested that systematic resistance to estrogen leads to obesity and increased body mass index; they presumed that this mechanism contributes to choroidal nevus onset. Studies of postmenopausal women also revealed that the incidence of choroidal nevi was twofold greater in overweight women than in normal-weight women. Vitamin C has been reported to reduce the incidence of choroidal nevi (22).

However, when the mass thickness is > 2 mm, as measured by ultrasound, the hazard ratio significantly increases for transformation from a choroidal nevus to a choroidal melanoma (25). Additional risk factors include worse Snellen visual acuity, empty echo findings on ultrasound, LBD > 5 mm on fundus examination, subretinal fluid on optical coherence tomography (OCT) examination, and orange pigment deposition (i.e., lipochrome) on autofluorescence examination (26–29). Traditional angiography methods (e.g., fluorescein angiography) are important for differentiating a choroidal nevus from a choroidal melanoma on the basis of subretinal vessel morphology. Current OCT technology is suitable for observation of vascular morphology information. When a choroidal nevus exhibits long-term exudative subretinal fluid and multiple punctures, OCT angiography (OCTA) shows choroidal neovascularization, consistent with fluorescein angiography findings (24, 30). Swept-source OCT can show narrow border vessels significantly related to the subretinal fluid (31); enhanced depth imaging (EDI)-OCT has demonstrated thin blood vessels covering 94% of choroidal nevi (32). Francis et al. (31) proposed that the tumor diameter is closely related to secondary retinopathy, while OCT can distinguish a choroidal nevus by identifying fine retinal structure. OCTA can reveal that the Bruch’s membrane–retinal pigment epithelium–Bruch’s membrane complex is complete and regular in a choroidal nevus, while the Bruch’s membrane–retinal pigment epithelium–Bruch’s membrane complex and the shape of the outer retinal layer are fuzzy in a choroidal melanoma (33). Notably, EDI-OCT has shown that photoreceptor cells exposed to subretinal fluid in a choroidal nevus exhibit atrophy (i.e., “stalactites” or “cracks”), while photoreceptor cells in a small melanoma became loose and exhibit “furry” morphology, with more irregular retinal layers accompanied by considerable structural damage (22, 26, 32). OCTA can distinguish the macular characteristics of choroidal nevi and choroidal melanomas; Valverde et al. suggested that the mass thickness is closely related to the difference of macular characteristics. The superimposed macular microvascular changes are smaller in choroidal nevi (34); the central macular thickness, fovea avascular area, and choroidal capillary thickness are similar to those parameters in healthy fellow eyes. In choroidal melanomas, central macular thickness increases, fovea avascular area increases, and choroidal capillary thickness decreases; moreover, the blood flow rate is considerably lower than normal (11.2%) (33).

## Diagnosis of Choroid Melanoma

For large choroidal melanomas, the diagnosis is mainly performed by slit lamp examination, indirect ophthalmoscopy, fluorescein angiography, and ultrasound (35). For early small choroidal melanomas, there is controversy regarding the most suitable diagnostic approach. Kivela et al. reported that biopsy should be performed to distinguish this type of tumor from a choroidal nevus (35), whereas Singh et al. stated that fine needle aspiration cytology should not be used due to the risk of tumor spread (3). Therefore, imaging is particularly important for the diagnosis of choroidal melanomas. Currently available imaging methods for choroidal melanomas include ultrasound (A-mode, B-mode, and color Doppler), magnetic resonance imaging (MRI), and OCT (i.e., swept-source OCT, spectral domain OCT, EDI-OCT, and OCTA). Each approach has unique advantages and disadvantages in various situations.

Since choroidal melanomas usually appear as low reflectivity, A-mode ultrasound can provide the best imaging effect (26). In A-mode ultrasound, choroidal melanomas exhibit low echo, smooth attenuation, and vascular pulsation (3). In B-mode ultrasound, choroidal melanomas can appear to be bulging. If the tumor penetrates Bruch’s membrane, it will assume a collar button or mushroom-like appearance; such tumors have no echo and contain a cavity in the posterior wall. These tumors also contain a choroid gap and orbital shadow (3, 36). Real-time high-resolution ultrasound (i.e., fusion ultrasound) can demonstrate choroidal melanoma and optic nerve structures in real time. Additionally, MRI combined with real-time color Doppler ultrasound can observe the tumor structure and evaluate the retrobulbar vascular system (37). Ultrasound is considered the most important method for evaluation of choroidal melanoma progression; ultrasound is superior to MRI in the detection of extrascleral extension (38, 39). When a choroidal melanoma involves the optic nerve, the fundus may have a similar manifestation of optic neuritis; MRI may show the tumor as a mass adjacent to the optic nerve. However, ultrasound evaluation can show the absence of an echo and reveal intraocular components, thus aiding in diagnosis of the mass (40). Nevertheless, for small choroidal melanomas, the specificity of ultrasound involving subretinal fluid is low, because such tumors cannot be distinguished from retinal thickening, cystic changes, and pigment epithelial detachment (41). EDI-OCT can partially compensate for this deficiency (36, 42).

MRI has limited diagnostic value for choroidal melanomas (3). For large tumors or those with poorly reflective structures in ultrasound examination, MRI can be used to detect whether the mass has penetrated through the sclera (36). However, the presence of nonspecific inflammation, angiogenesis, and motion artifacts can lead to false positive results (38).

OCT can reveal extensive details of choroidal melanomas. In particular, this method can show whether the choroidal melanoma surface is regular and lobular (43), whether it exhibits normal retinal thickness and a complete photoreceptor cell layer, and whether extensive retinal detachment and “debris” are present in the dorsal retina (19). Swept-source OCT can show fine details of the choroid near the fovea and posterior pole (44). Furthermore spectral domain OCT can help to observe lesions in the pigment epithelium (45). The combined use of OCT and fundus fluorescein angiography can help to identify subretinal fluid and orange pigment (35). Previous studies have shown that 60% of the subretinal fluid in a choroidal melanoma is patchy, while 40% is diffuse (46). EDI-OCT is an effective tool to identify structural changes in the retina in patients with small choroidal melanomas (47). This method can show retinal edema, “fluffy” or lost photoreceptor cells, an irregular ganglion cell layer, broken connections between internal and external segments, an irregular inner plexus layer, the loss of external membrane, and other structural damage (26). It is also more accurate for measurement of tumor thickness, compared with ultrasound. Specifically, the tumor thickness is 55% greater when measured by ultrasound than when measured by EDI-OCT, which reveals the most actual thickness. Additionally, EDI-OCT can identify a subclinical level of peripheral subretinal fluid that is not yet visible *via* ophthalmoscopy (36). Therefore, EDI-OCT is important in the early diagnosis and evaluation of choroidal melanomas.

In addition to the retinal structure, advancements in OCT technology have improved the visibility of the tumor vasculature. Large choroidal melanomas have a double cycle; extensive and progressive fluorescence are evident under fundus fluorescein angiography (3). Furthermore, swept-source OCT can clearly display the intrinsic tumor vasculature, with an effect similar to that of indocyanine green angiography (48). With the exception of hemangiomas, most choroidal tumors show internal compression of the vasculature (45). Therefore, OCTA shows that choroidal melanomas have a dense and irregular vascular network in the outer retinal layer and choroidal layer, while choroidal nevi show reduced blood flow in the corresponding area (49).

## PET/CT to Predict Recurrence or Metastasis of Choroidal Melanoma

As mentioned above, early melanocyte aggressiveness is closely related to tumor prognosis. Therefore, evaluation of the risks of choroidal melanoma recurrence and metastasis on the basis of early cytological behavior can provide timely prognostic information; this can be combined with histological evaluation of the tumor to provide more guidance for medical decision-making. PET/CT is appropriate for this application; it can aid in early intervention before morphological recurrence and tumor metastasis, thus greatly improving patient survival.

PET/CT is typically used to evaluate tumor activity and the risks of recurrence or metastasis by means of cell metabolism assessment. Metabolic activity is negatively correlated with metastasis duration (50). Parameters related to cell metabolism include maximum standardized uptake value (SUVmax) and metabolic rate of glucose. SUVmax is defined as the ratio between the radiation concentration and the injection dose in the region with the highest uptake signals of ^18^F-fluorodeoxyglucose (^18^F-FDG, the analog of glucose) and other tracers; this parameter can be used to semi-quantitatively evaluate cellular metabolic rates (51). The metabolic rate of glucose is the product of ^18^F-FDG clearance rate and blood glucose concentration (i.e., glucose uptake rate per unit of tracer distribution) (52). This parameter can accurately quantify the degree of metabolic activity; it also helps to distinguish among tumor cell types, thereby indirectly evaluating the risks of recurrence and metastasis. Notably, epithelioid cell melanomas have worse prognoses than spindle cell melanomas (53). Similarly, SUVmax and LBD are significantly associated with metastatic death. Higher SUVmax is reportedly indicative of greater tumor diameter or thickness (54). Among patients with choroidal melanoma who underwent 6 months of treatment, choroidal thickness was significantly reduced, compared with baseline (55). Therefore, the magnitude of SUVmax may influence the therapeutic effect. Furthermore, the magnitude of SUVmax is reportedly related to the pathological classification of melanoma. Higher SUVmax often indicated a nodular tumor, while lower SUVmax implied diffuse infiltration (54). Chromosome 3 monosomy and chromosome 8 amplification are strongly correlated with choroidal melanoma metastasis. The metastasis and prognosis of choroidal melanoma are mainly related to the above cytogenetic changes, rather than the AJCC stage. Notably, some studies have suggested that SUVmax is related to chromosome 3 monosomy in choroidal melanomas. Among those tumors, 92% have SUVmax > 2.5, while 67% have SUVmax > 4. Therefore, SUVmax > 4 may be an indirect indicator of chromosome 3 monosomy in choroidal melanomas (56).

PET can qualitatively assess tumor development by monitoring variations in cell metabolism. Surveillance with ^18^F-FDG PET/CT may exclude metastasis or suggest necrotic choroidal melanoma if cell metabolism is obviously low. Clinically significant changes in the metabolic activity of a lesion may indicate local recurrence (57, 58).

## Combined Multimodal and Molecular Imaging to Assess Metastasis of Choroidal Melanoma

### Routine Detection of Liver Metastasis

The liver is the most common metastatic site of choroidal melanomas (59), such that approximately half of affected patients have liver metastasis (60). Moreover, liver metastases are often the first non-ocular locations affected (61). The median disease-free survival time of patients with choroidal melanoma is 26 months, while the median survival time is only 8 months after initial metastasis detection (62). Patients often die of liver failure, manifested by ascites and hepatomegaly. Biopsy of metastases revealed a maximum size of 100 cm^2^ (63). Presumably, the most important factors influencing survival rate are the levels of liver enzymes, especially lactate dehydrogenase and alkaline phosphatase (64). Mariani et al. have found that if LDH is 1.5 times higher than normal, it indicates poor prognosis of the patient (65). If these levels have been normal and the disease-free interval is > 36 months, the overall survival period is expected to be considerably longer (62). Therefore, early treatment of metastasis and management of liver enzyme levels are important considerations for patient survival.

Serial hepatic ultrasound and confirmatory scans (e.g., CT) can reveal asymptomatic metastasis (66). However, Mariani et al. have suggested that USG can only detect lesions on the liver surface, which indicated the limited effect (65). For newly diagnosed uveal melanoma, MRI staging can accurately recognize early liver metastasis (61, 67). Mariani et al. believe that the number and maximum surface area (>800mm^2^) of metastasis under MRI can be used as important indicators to predict the survival rate of patients (65). Because of the vascular richness in uveal melanoma, a metastasis on MRI appears as multiple enhanced solid liver lesions with the high T1 signal characteristic of melanomas (68). Diffuse-weighted images (DWI) can be used to detect more metastasis, which appearances as the high signal, and the lesion can still exist when the dispersion sensitivity coefficient is adjusted to the highest, thus more metastasis < 5mm can be detected, regardless of the location of the metastasis in the liver (65). Regarding the frequency of MRI detection, intervals of 3 months (60) and 6 months (69) have been suggested.

In CT scans, liver metastases often constitute multiple, heterogeneous, hypodense, and enhanced lesions, with an average size of 46.8 cm^2^ (63). The detection efficiency of dual-energy CT with low kVp is superior to that of virtual 120 kVp CT with digital subtraction angiography images (70). Positive CT or MRI findings were compared with positive ultrasound findings; the results showed that 53% of the CT/MRI findings were completely consistent with ultrasound findings, 11% were completely inconsistent, 29% were negative findings on ultrasound, and 7% could not detect the positive findings on ultrasound. It has been suggested that CT scans of the chest, abdomen, and pelvis should be performed before the assessment of the patient’s prognosis (71). Thus, CT/MRI is presumed to constitute a more efficient approach than ultrasound; in particular, confirmatory MRI scanning may be useful in patients with abnormal liver enzyme levels (72).

### Usefulness of PET Staging for Choroidal Melanomas

According to the AJCC, choroidal melanomas are divided into the following four stages: T1, LBD < 10 mm and thickness < 2.5 mm; T2, LBD 10–16 mm and thickness 2.5–10 mm; T3, LBD > 16 mm and thickness > 10 mm, without extraocular invasion; T4, LBD > 16 mm and thickness > 10 mm, with extraocular invasion. T1 and T2 stages are each divided into three clinical sub-stages on the basis of the external invasion status: none, microscopically visible, and visible with the unaided eye. Thus, extraocular invasion status (i.e., extraocular extension and metastasis) is important for the staging of choroidal melanomas.

Although PET/CT is not currently used as a routine method for detection of metastasis, it can be used to detect metastasis in patients with normal CT findings and normal liver enzyme levels (73, 74). Thus, this approach is helpful for metastasis screening and tumor staging; it can also be used as a supplementary alternative for patients with contraindications to MRI (75). Most liver metastases can be qualitatively detected through PET/CT, in combination with ^18^F-FDG to detect local uptake and MRI for confirmatory diagnosis (76, 77). Furthermore, PET/CT is suitable for detecting extrahepatic metastases of T4 stage tumors (78). Second primary cancers in 10% of patients with choroidal melanomas can be detected by PET/CT (77). However, the initial staging value of PET/CT for choroidal melanoma is not superior to MRI (76, 79), because PET/CT can only detect 33% of T2 stage tumors and 75% of T3 stage tumors (80). Small liver lesions cannot be detected by PET/CT (59, 79), while MRI of the abdomen and chest can detect nearly all metastases of uveal melanomas (81). In addition to lung and liver metastases, PET/CT can detect suspected local uptake of ^18^F-FDG in lymph nodes, which can then be confirmed by ultrasound and fine needle aspiration cytology (58). Therefore, ultrasound combined with PET/CT has been proposed for the initial staging of choroidal melanomas; chest radiography combined with analysis of liver enzyme levels may be appropriate for long-term staging (77).

**Figure 1:**
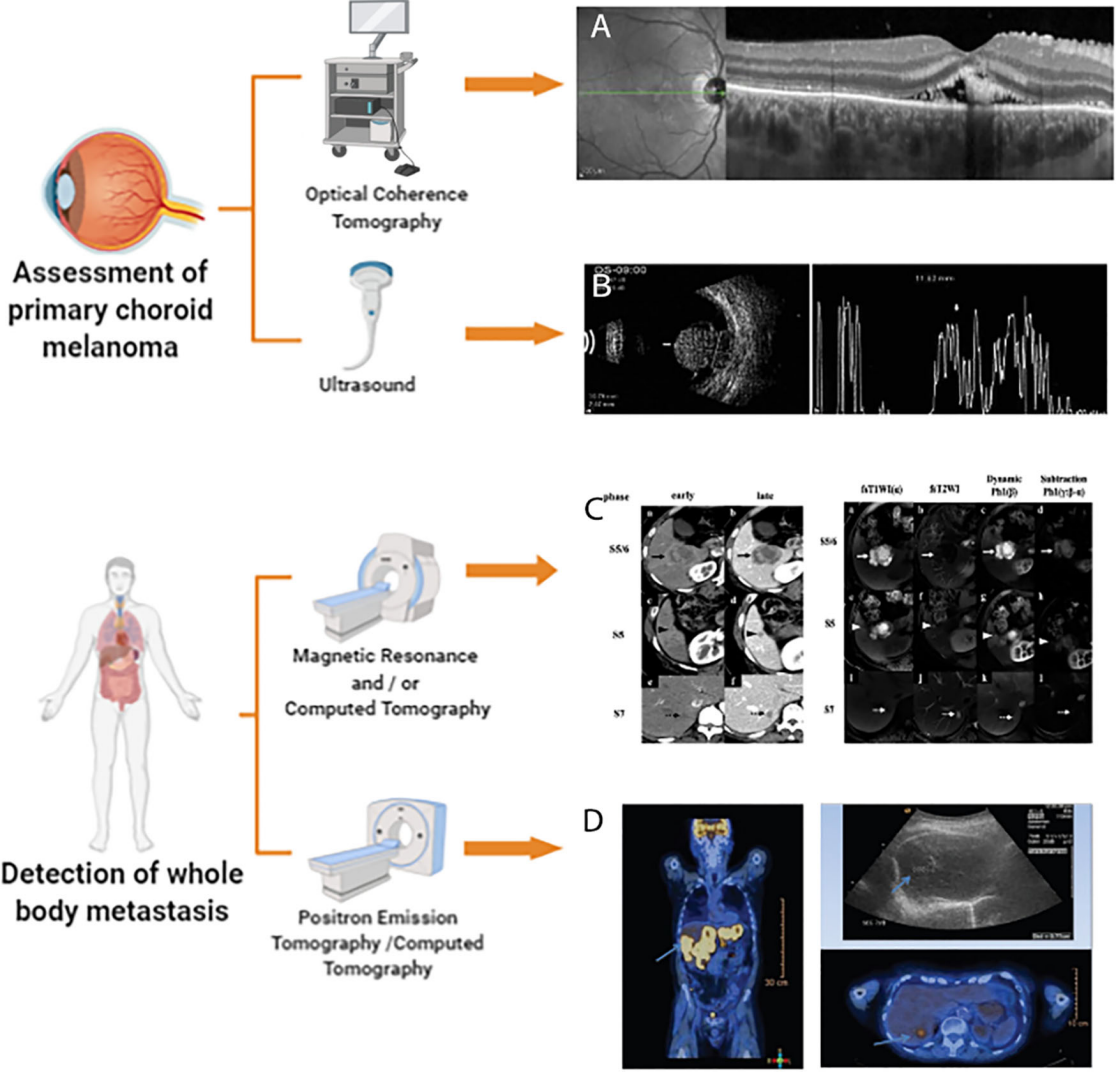
Multimodal and Molecular imaging techniques to assess choroidal melanoma. This illustration is created by Biorender.com **(A)** enhanced depth imaging (EDI)-OCT image is reprinted with permission from ref (42).Copyright ^©^ 2015 Ann Q. Tran et al. **(B)** Ultrasound image is reprinted with permission from ref (3).Copyright ^©^ 2012 Singh P and Singh A **(C)** magnetic resonance imaging (MRI) and CT image is reprinted with permission from ref (66).Copyright ^©^ 2020 The Author(s) **(D)** PET/CT image is reprinted with permission from ref (75).Copyright ^©^ 2018 Middle East African Journal of Ophthalmology.

Because PET/CT can assess cell metabolism and anatomical changes throughout the body, it is applicable for use in all patients with choroidal melanoma who require evaluation and/or restaging of extrahepatic metastases (82). **Figure 1** accounts for the current multimodal and molecular imaging techniques assessing the primary tumor and metastasis of choroidal melanomas.

## Discussion

Choroidal melanomas are insidious tumors prone to extraocular extension and metastasis. Therefore, early diagnosis of choroidal melanomas and timely detection of metastases are particularly important. Early choroidal melanomas can easily be confused with choroidal nevi, so accurate identification of choroidal nevi is needed. Pathological examination is considered the gold standard for cancer diagnosis; thus far, invasive examination is not recommended for early choroidal melanomas. Therefore, non-invasive imaging methods are critical for the assessment of patients with suspected choroidal melanoma. Ultrasound is an important method for the diagnosis and monitoring of choroidal melanomas; in combination with other imaging techniques, ultrasound enables real-time observation of tumors and their posterior structures. Compared with ultrasound, OCT methods (especially EDI-OCT) provide more accurate and detailed observations of the morphology and blood supply of choroidal melanomas, offering valuable information for the diagnosis and monitoring of these tumors. MRI and CT are of limited value during diagnosis of the primary tumor, but are indispensable for tumor staging and metastasis detection. PET/CT is a powerful supplement to the above imaging methods for the staging of choroidal melanomas; it can identify metastases that not detected in a timely manner by conventional imaging methods.

Current technology allows the use of PET/CT to assess the risks of tumor recurrence and metastasis at the cytological level, prior to histological changes, by monitoring the metabolic behavior of melanoma cells. This information provides a reference for medical decisions and has the potential to greatly improve the survival and quality of life for affected patients. At the same time, there is an unavoidable challenge that the use of SPECT (such as ^123^IMP-SPECT) has the higher detection rate than ^18^FDG-PET/CT (83). Therefore, more effective radioactive tracer should be actively sought for PET/CT imaging of choroidal melanoma, so as to improve the sensitivity of PET/CT to the detection of micro-liver metastasis.

## Author Contributions

The co-first authors of this article, XL and LW, were responsible for the conception and design of the manuscript, and conducted data collection and analysis, made valuable and constructive changes to the content of the manuscript, confirmed the final version of this article, and agreed responsible for all matters related to the publication of this manuscript. The corresponding author, XW, who is in charge of the publishing process and established the core idea of this manuscript. Two co-authors, LZ and FT, provided many valuable suggestions and sources and made some crucial amendments to the manuscript. All authors contributed to the article and approved the submitted version.

## Funding

This work was supported by grants from the Natural Science Foundation of China (No.82070954), The Applied Basic Research Programs of Science and Technology Commission Foundation of Sichuan Province (No.19YYJC0790), and The Innovative Spark Grant of Sichuan University (No.2018SCUH0062).

## Conflict of Interest

The authors declare that the research was conducted in the absence of any commercial or financial relationships that could be construed as a potential conflict of interest.
